# Burn injury characteristics: findings from Pakistan National Emergency Department Surveillance Study

**DOI:** 10.1186/1471-227X-15-S2-S5

**Published:** 2015-12-11

**Authors:** Emaduddin Siddiqui, Nukhba Zia, Asher Feroze, Safia Awan, Arifa Liaquat Ali, Junaid Abdul Razzak, Adnan A Hyder, Asad Latif

**Affiliations:** 1 Department of Emergency Medicine, Aga Khan University, Karachi, Pakistan; 2 Department of Medicine, Aga Khan University, Karachi, Pakistan; 3 Department of Emergency Medicine, John Hopkins School of Medicine, Baltimore, MD, USA; 4 Johns Hopkins International Injury Research Unit, Department of International Health, Johns Hopkins Bloomberg School of Public Health, Baltimore, MD, USA; 5 Department of Anesthesiology and Critical Care Medicine, Johns Hopkins University School of Medicine, Baltimore, MD, USA; 6 The author was affiliated with the Department of Emergency Medicine, Aga Khan University, Karachi, Pakistan at the time when study was conducted

**Keywords:** Burns, surveillance, Pakistan

## Abstract

**Background:**

Burn injury is an important yet under-researched area in Pakistan. The objective of this study was to determine the characteristics and associated outcomes of burn injury patients presenting to major emergency departments in Pakistan.

**Methods:**

Pakistan National Emergency Department Surveillance (Pak-NEDS) was a pilot active surveillance conducted between November 2010 and March 2011. Information related to patient demographics, mode of arrival, cause of burn injury, and outcomes was analyzed for this paper. Data were entered using Epi Info and analyzed using SPSS v.20. Ethical approval was obtained from all participating sites.

**Results:**

There were 403 burn injury patients in Pak-NEDS, with a male to female ratio 2:1. About 48.9% of the burn injury patients (n = 199) were between 10 - 29 years of age. There was no statistically significant difference between unintentional and intentional burn injury patients except for body part injured (p-value 0.004) and ED disposition (p-value 0.025). Among 21 patients who died, most were between 40 - 49 years of age (61.9%) and suffered from fire burns (81%).

**Conclusion:**

Burn injuries are a burden on emergency rooms in Pakistan. We were able to demonstrate the significant burden of burn injuries that is not addressed by specialized burn centers.

## Background

Burn injuries are a global public health problem with physical, psychological, and economic implications for patients, their families, and society as a whole [1]. Around 90% of all burns injuries occur in low- and middle income countries (LMICs) [2]. Burns are the fourth leading cause of injury following road traffic injuries, falls, and interpersonal violence, accounting for 5 - 12% of all injuries worldwide and around 11 million patients requiring medical attention [3-5]. About 265,000 people die each year due to burn injuries according to the World Health Organization (WHO) [6]. This current burden of burn injuries is deeply inequitable, with incidence disproportionately affecting the poor and the vulnerable [7].

Around two-thirds of burn injuries occur in the African, Eastern Mediterranean and South-East Asia regions of the WHO [8]. The annual incidence of burn injuries in the Eastern Mediterranean and South-East Asia regions is estimated to be 187 and 243 per 100,000 population, respectively [9]. Mortality rates from fire-related burns are the highest in the South-East Asia region (11.6 per 100,000 population) and when compared to the 1 death per 100,000 population found in high-income countries (HICs), this is one of the largest discrepancies for any injury mechanism [2]. The pattern of burn injury and the groups affected in LMICs and HICs also differs; for example, among the 15-59 year age group, the mortality rate due to fire-related burn injury in HICs is twice as high for males compared to females, while the reverse is true in LMICs [2].

Burn injury is an important yet under-researched area in Pakistan. The Global Burden of Disease 2010 study estimates that the age-standardized mortality rate for injury caused by fire, heat, and hot substances is 5.8 per 100,000 population in Pakistan [2]. A burn facility-based study from Karachi estimated burn-associated mortality rate among adults between 15 - 55 years to be even higher at 10.2 per 100,000 population [10]. Some risk factors reported to be associated with admission and mortality include female gender, age >50 years, fire burn, inhalational injury, and total body surface area (TBSA) of >40% [11,12]. However, most of the existing knowledge regarding burn injuries in Pakistan is from single-center studies in specialized burn hospitals found in major cities [11-15].

Existing data make it difficult to estimate the true burden of burn injuries on the general population and little is known about the epidemiology of burn injury in the population that does not present to specialized burn centers. This is an important knowledge gap as specialized burn centers are not accessible to a large portion of the Pakistani population. Emergency departments (EDs) are a logical first point of contact for care of burn injury patients. The overall aim of this study is to determine the characteristics of burn injury patients presenting to urban tertiary care EDs across Pakistan. The specific objectives of the study are to understand burns as a mechanism of injury for unintentional and intentional injuries and to look at burn injury characteristics based on patient disposition from the ED.

## Methods

The Pakistan National Emergency Departments Surveillance (Pak-NEDS) was an active surveillance program conducted in seven major urban tertiary care EDs across Pakistan. Data was collected between November 2010 and March 2011 on all patients seeking care in participating EDs. Pak-NEDS enrolled 274,436 patients. The study sites consisted of two private and five public sector hospitals, each of which are considered major tertiary care centers in their respective cities. These centers included: the Aga Khan University (AKU) and Jinnah Post-graduate Medical Center in Karachi; Benazir Bhutto Hospital in Rawalpindi; Lady Reading Hospital in Peshawar; Mayo Hospital in Lahore; Sandeman Provincial Hospital in Quetta; and Shifa International Hospital in Islamabad.

A one-page standardized data collection tool was developed based on the ambulatory care survey from the Centers for Disease Control and Prevention, USA and previous surveillance conducted in Pakistan [16,17]. The tool was reviewed and approved by the heads of the EDs at each of the institutions involved in the study. Data were collected by dedicated data collectors hired and trained specifically for Pak-NEDS. All patients coming to the participating EDs were approached after their registration in the ED; data was gathered by interviews with patients or next of kin, from the ED physician, and by review of the patient medical record. All data were gathered while the patient was in the ED and there was no contact with the patient following discharge from the ED. Ethical approval was obtained from all participating sites in Pakistan as well as from the Institutional Review Board of the Johns Hopkins Bloomberg School of Public Health in the United States.

For the purpose of this analysis, we looked at all the patients in the Pak-NEDS dataset who presented to the ED with any form of burn injury. We analyzed information related to patient demographics (such as age, gender, and ethnicity), mode of arrival to ED, cause and intent of burn injury, and disposition from the ED. Data was entered into Epi Info version 3.3.2 by a data management team at AKU [18]. For purposes of comparison, age was categorized into seven age groups: under 5 years, 5 - 9 years, 10 - 19 years, 20 - 29 years, 30 - 39 years, 40 - 49 years, and 50 years and above. Patient disposition from the ED was categorized into: discharged home from ED; admitted to hospital for further care and management; death in ED; or 'Other,' which included left ED without being seen, left against medical advice, or referred to another facility. Data analysis was conducted using SPSS version 20 [19].

For descriptive analysis, frequencies for gender, type of hospital, mode of arrival to ED, intent of injury, and cause of injury were calculated. Due to the non-normal distribution of age in the dataset, median age and interquartile range were calculated. The intent of burn injury and three disposition categories - discharged from ED, admitted, and death in ED - were separately compared with patient demographic and burn injury characteristics using chi squared tests and Fisher Exact test. The level of significance was set at 0.05.

## Results

There were 403 burn injury patients in Pak-NEDS. This translates into 146.8 burn injury patients per 100,000 ED visits in Pakistan. Males comprised 67.2% (n = 271) of patients, outnumbering females in all age groups leading to an overall male to female ratio of 2:1 (Table 1). Nearly half of the patients (n = 199) were between 10 - 29 years of age; a quarter of the patients were over 40 years. The median age was 25 years (interquartile range 19 - 38 years). Most burn injury patients arrived to the ED via non-ambulance transport (n = 331, 82.13%).

**Table 1:** Demographic characteristics of burn patients in Pakistan (n = 403)

Demographic Characteristics	n (%)
**Gender**	
Male	271 (67.2)
Female	132 (32.8)

**Age Group**	
Under 5 years	22 (5.5)
5-9 years	19 (4.7)
10-19 years	68 (16.9)
20-29 years	131 (32.5)
30-39 years	66 (16.4)
40-49 years	59 (14.6)
≥ 50 years	38 (9.4)

**Mode of Transport**	
Ambulance	25 (6.2)
Non ambulance	331 (82.13)
Not reported	47 (11.7)

**Hospital Type**	
Public Hospital	377 (93.5)
Private Hospital	26 (6.5)

The intent of the injury was known for about three-quarters (n = 308) of burn patients and of these about 65.3% (n = 201) were reported to be unintentional in nature. For about a quarter of patients (n = 107, 26.5%), burns were recorded as intentional burns. The main causes of burns were scalds (n = 259, 64.3%), followed by fire, flame, or smoke burns (n = 66, 16.4%). The injured body region was known in 320 patients (79.4%), with extremities being the most commonly burnt (n = 174, 54.4%), followed by head, neck, and face (n = 101, 31.6%). Patient disposition from the ED was known for about 80% (n = 324) of patients; 20.1% (n = 65) were admitted for further care, and 6.5% (n = 21) died in the ED (Table 2).

**Table 2:** Burn injury characteristics in Pakistan (n = 403)

Burn Injury Characteristics	n (%)
**Intent of injury (n = 308)**	
Self-inflicted	45 (14.6)
Assault	62 (20.1)
Unintentional	201(65.2)

**Cause Of Burn**	
Scalds	259 (64.3)
Fire burns	66 (16.4)
Chemical	10 (2.5)
Others	68 (16.9)

**Body Part Injured (n = 320)**	
Limbs	174 (54.4)
Head, neck and face	101 (31.6)
Thorax	20 (6.3)
Other	25 (7.8)

**ED Disposition (n = 324)**	
Discharged from ED	218 (67.3)
Admitted	65 (20.1)
Expired	21 (6.5)
Others *	20 (6.2)

*Includes referral and left against without being seen

Missing data: 23.6% for intent of injury, 20.6% for body part injured, 19.6% for ED disposition

The intent of burn injury was compared with patient demographic (gender, age group, mode of transportation, hospital type) and burn injury characteristics (cause of burn, body part injured, degree of burns, ED disposition). There was no statistically significant difference between unintentional burn injury patients and patients with intentional injury caused by burns except for body part injured (p-value 0.004) and ED disposition (p-value 0.025). (Table 3)

**Table 3:** Comparison of characteristics of burn injury patients based on intent of injury (n = 308)

Variables	Intent of injury				p-value*
		
	Unintentional n = 201 (65.2%)		Intentional n = 107 (34.7%)		
		
	n	%	n	%	
**Gender**					0.343
Male	138	68.7	79	73.8	
Female	63	31.3	28	26.2	

**Age Group**					0.097**
Under 5 years	14	7	5	4.7	
5-9 years	15	7.5	3	2.8	
10-19 years	32	15.9	16	15	
20-29 years	67	33.3	37	34.6	
30-39 years	26	12.9	25	23.4	
40-49 years	32	15.9	10	9.3	
≥ 50 years	15	7.5	11	10.3	

**Mode of Transport (n = 269)**					0.135
Ambulance	10	5.8	12	12.2	
Non ambulance	161	94.1	86	87.8	

**Hospital Type**					0.106**
Public Hospital	181	90	102	95.3	
Private Hospital	20	10	5	4.7	

**Cause Of Burn**					0.620**
Scald	135	67.2	64	59.8	
Fire/Flame/Smoke	32	15.9	21	19.6	
Chemical	5	2.5	4	3.7	
Others	29	14.4	18	16.8	

**Body part injured (n = 278)**					
Limbs	89	48.4	49	64.5	
Head, neck and face	60	32.6	22	28.9	0.004**
Thorax	10	5.4	5	6.6	
Other	25	13.6	0	0	

**Degree Of Burn (n = 39)**					0.147**
>20%	21	61.8	1	20	
<20%	13	38.2	4	80	

**ED disposition (n = 252)**					0.025**
Discharged from ED	109	64.9	69	82.1	
Admitted from ED	28	16.7	10	11.9	
Death	17	10.1	3	3.6	
Others***	14	8.3	2	2.4	

*p-value: bold values are significant. Analysis was conducted using Chi square test with level of significance 0.05

** Analysis was conducted using Fisher Exact test with level of significance 0.05

***Left without being seen, left against medical advice, referred patients

Table 4 compares patient and burn injury characteristics based on three ED disposition groups (discharged from ED, admitted, and death). A statistically significant difference was observed in all patient and burn injury characteristics between the three categories of ED disposition except for intent (p-value 0.186) and degree of burns (0.073). Patients between 20 - 29 years of age comprised the largest proportion of those discharged from the ED (36.7%, n = 80) and those that were admitted (30.8%, n = 20). Out of the 21 patients who died in the ED, 61.9% were between 40 - 49 years of age. There were no deaths in children under 10 years of age. The majority of patients who were discharged home from the ED (n = 212, 97.2%) or were admitted (n = 63, 96.9%) presented to the EDs of public hospitals while the majority of those who died due to burns presented to private hospitals (n = 16, 76.2%) (p-value <0.001).

**Table 4:** Comparison of ED disposition of burn injury patients (n = 304)

Variables	ED disposition of burn injury patients						p-value*
		
	Discharged from ED n = 218		Admitted n = 65		Death n = 21		
		
	n	%	n	%	n	%	
**Gender**							**0.019**
Male	152	69.7	38	58.5	19	90.5	
Female	66	30.3	27	41.5	2	9.5	

**Age Group**							**<0.001**
Under 5 years	11	5	5	7.7	-0	0	
5-9 years	11	5	3	4.6	0	0	
10-19 years	39	17.9	10	15.4	2	9.5	
20-29 years	80	36.7	20	30.8	3	14.3	
30-39 years	32	14.7	14	21.5	3	14.3	
40-49 years	27	12.4	7	10.8	13	61.9	
≥ 50 years	18	8.3	6	9.2	0	0	

**Mode of Transport (n = 270)**							**0.04**
Ambulance	11	5.3	7	11.7	1	33.3	
Non ambulance	196	94.7	53	88.3	2	66.6	

**Hospital Type**							**<0.001**
Public Hospital	212	97.2	63	96.9	5	23.8	
Private Hospital	6	2.8	2	3.1	16	76.2	

**Intentional Type**							0.186
Self-inflicted	33	18.5	4	10.5	1	5	
Assault	36	20.2	6	15.8	2	10	
Unintentional	109	61.2	28	73.7	17	85	

**Cause Of Burn**							**<0.001**
Scalds	153	70.2	36	55.4	1	4.8	
Fire/Flame/Smoke	29	13.3	8	12.3	17	81	
Chemical	6	2.8	1	1.5	1	4.8	
Others	30	13.8	20	30.8	2	9.5	

**Body part injured (n = 252)**							**<0.001**
Limbs	113	63.1	26	48.1	0	0	
Head, neck and face	53	29.6	22	40.7	1	5.3	
Thorax	11	6.1	6	11.1	1	5.3	
Other	2	1.1	0	0	17	89.5	

**Degree Of Burn (n = 26)**							0.073
>20%	7	36.8	6	85.7	0	0	
<20%	12	63.2	1	14.3	0	0-	

*p-value: bold values are significant.

Analysis was conducted using Fisher Exact test with level of significance 0.05

Burns caused by scalds were the most common reason amongst those discharged from the ED (n = 153, 70.2%), followed by fire, flame, or smoke (n = 29, 13.3%). The common reasons amongst those admitted from the ED were scald (n = 36, 55.4%). Fire, flame, or smoke was the most common cause of burns in patients who died in the ED (n = 17, 81%). The extent of the burn injury was known for only a very small number of patients (n = 26); a vast majority of those who were admitted suffered from >20% burns (n = 6, 85.7%). Information regarding degree of burns for patients who died was not available.

## Discussion

This study presents data from seven major tertiary care centers in Pakistan, capturing important characteristics of burn injury patients. It is one of few studies to show the actual burden of burn injuries on the healthcare system. The majority of these patients used public transport to access the healthcare system. We show that males and young adults experience a higher proportion of burn injuries, but the majority of the mortality burden is borne by those older than 40 years. A high proportion of burn injury patients presenting to private hospitals died. Hot surfaces or liquid and burns due to fire were the most common causes of burns, with death occurring more frequently among patients with fire-related burn injuries.

Our calculated incidence rate of 147 per 100,000 patients presenting to Pakistani EDs is almost twice that noted in a previous study conducted in Rawalpindi, Pakistan which reported an incidence of 76 per 100,000 for ED visits [20]. Our observations on gender differed from other studies from Pakistan, India, and Eastern Mediterranean region, with fewer females as compared to males experiencing burn injuries [11,14,21,22]. The reason for fewer females presenting with burn injuries in our study may be due to under-reporting in females, delay in seeking care. For example, women might not seek care, or not be supported in seeking care, especially if the injury was intentional. This study shows a higher percentage of men presenting with intentional burn injuries. This is in contrast to a recent study from Nepal which reported more females presenting with intentional burn injury [23]. Our findings also differ from various facility-based studies from India and China [22,24] and population-based reviews from the Eastern Mediterranean and South-East Asia regions of WHO [21,25], where males under 5 years and adolescent and young females were reported to experience more burn injuries.

Similar to this study, a higher incidence of burn injuries among young adults has been reported at the country and regional level in other studies from China, Pakistan, India, Iran, and Turkey [15,21,22,24-27]. However, other studies from Bangladesh and Oman have identified children and elderly as the most vulnerable groups, which is in contrast to this study's findings [7,28,29]. Injuries in young adults have economic implications for both the affected individuals and their families, given high levels of participation in the workforce at these ages, necessitating an accurate assessment.

In our study, those who died had suffered from fire-related burns and were more likely to be older individuals. Burn-related injuries are the fourth leading cause of unintentional injury death in the elderly and this risk is disproportionately higher for this age group compared to the general population [6]. Our study showed overall burn mortality to be 6.5%, which is lower compared to other studies performed in Pakistan, which have reported mortality due to burn injuries ranging from 14% - 41.3% [11-15]. A possible reason for this discrepancy could be the fact that several of these earlier studies were conducted in specialized burn centers and units and/or that there may be under-reporting of deaths in Pak-NEDS. The mortality due to burn injuries among various countries in the WHO Eastern Mediterranean Region varies between 5% - 37% [21]. This variation in mortality could be because of different study settings, burn severity, availability of experts, or resources to manage burns. Although previous studies on burns have shown that there is higher mortality among patients with intentional burn injuries, our study showed that the majority of such patients were discharged from the ED [23]. This could be because most of the patients with intentional burn injuries are taken to specialized burn centers due to the severity of their injuries.

Scald and flame burns were the most common cause of burns, which is consistent with previous studies from Pakistan, Iran, India, and reviews from the EMRO region [11,13,14,21,22,25,27,30]. Our study lacks information related to the cause of the fire or flame; however, previous studies have reported kerosene stove burners and wood-based cooking fuel as the main source of burn related injuries amongst females of Southeast Asia [22].

Most of the existing centers are located in major cities of the country. Karachi, the largest metropolis in the country with more than 20 million people, has just one public and one private sector burn center [11,31]. More rural areas of the country face an even worse situation, with few centers equipped to handle burn injuries and limited options for referral. These facilities, most often in the public sector, are typically preferred by patients, as they are economical or free of cost, and more easily accessible. However, they tend to lack basic necessities such as equipment and trained healthcare providers.

Our analysis demonstrated that most of the deaths by burn injury occurred in private hospitals, perhaps as a result of more severe burns being treated there compared to those who went to public hospitals. Another possible cause could be that these patients may have been referred to private hospitals from public hospitals for further management [15]. There are also other factors that may influence the lack of care for patients with burn injuries in Pakistan including: densely populated areas; the vast catchment area for each tertiary care hospital (public and private); poor economic and educational status of the population; lack of awareness regarding prevention practices, such as safe fire use; or use of non-approved and inappropriate first aid treatments like applying tooth paste, cow dung, or other home remedies [32,33].

This study was one of the largest multi-center collaboration of EDs in Pakistan; the hospitals represented only major tertiary care hospitals in large cities across the country. This study therefore might not have captured patients who do not typically present at these types of hospitals, such as those that might have utilized primary or secondary centers, or those who might have gone to dedicated burn centers. The urban locations of the participating hospitals might have also resulted in a skewed distribution of demographic characteristics of patients, burn injury, and outcome. This study lacked information related to location of burn injury such as home or workplace; causes of burn such as kerosene oil, electricity, or chemicals; severity of burn injury including total body surface area burnt; and associated injuries and comorbid conditions. These are important factors to consider to determine outcomes of burn injury patients [21].

The dataset had missing information for some variables such as 23.6% for intent of injury, 20.6% for body part injured, and 19.6% for ED disposition. This resulted in limitations in performing regression analysis to get an in-depth understanding of risk factors associated with the burns presenting to EDs in Pakistan. This highlights the need for better data records in the EDs of Pakistan.

## Conclusion

We were able to demonstrate a significant burden of burn injuries that is not addressed by specialized burn centers. There is also a critical need to continue research and surveillance on burn patients in Pakistan, in order to broaden the evidence base on the epidemiology of these injuries, as well as the outcomes of patients following discharge. Such information, if available in future studies, will inform programs and policy emphasizing primary prevention, short-term treatment, and long-term management of burn injuries in low-resource settings such as Pakistan where these efforts are extremely deficient.

## Competing interests

The authors declare that they have no competing interests.

## Authors' contributions

EMS developed the initial draft. NZ was involved in data analysis and interpretation, draft writing and revision of the manuscript. AF and SA were involved in data management and analysis. ALA helped with the writing of the draft. AL was involved with data analysis and interpretation, and critical revision of the manuscript. JAR and AAH were involved in design of Pak-NEDS, and had overseen overall data analysis and interpretation and review of the paper. All authors approved of the final draft.
